# Detecting changes in the blood flow of the optic disk in patients with nonarteritic anterior ischemic optic neuropathy *via* optical coherence tomography–angiography

**DOI:** 10.3389/fneur.2023.1140770

**Published:** 2023-03-24

**Authors:** Qing Xiao, Chuan-bin Sun

**Affiliations:** Eye Center, Second Affiliated Hospital of Zhejiang University School of Medicine, Hangzhou, Zhejiang, China

**Keywords:** vessel density (VD), visual field (VF), nonarteritic anterior ischaemic optic neuropathy, optical coherence tomography angiography, ganglion cell complex (GCC)

## Abstract

**Purpose:**

This study aimed to evaluate the changes in the blood flow of the optic disk in patients with nonarteritic anterior ischemic optic neuropathy (NAION) using optical coherence tomography–angiography (OCTA) and to investigate the relationship among the changes in the blood flow of the optic disk, visual field defect, peripapillary retinal nerve fiber layer (RNFL), and macular ganglion cell complex (mGCC).

**Methods:**

This was a prospective observational case series study. A total of 89 patients (89 eyes) with NAION were included in this study. All patients underwent best corrected visual acuity (BCVA), slit-lamp and direct ophthalmoscopic examinations, color fundus photography, visual field test, and blood flow indicators of the radial peripapillary capillaries (RPC) including whole en face image vessel density (VD), peripapillary VD by OCTA, the peripapillary RNFL, and mGCC by spectral-domain optic coherence tomography (OCT). The changes of blood flow in the optic disk at ≤3, 4–8, 9–12, 13–24, and >24 weeks of the natural course of NAION were measured, and the relationship among the changes in the blood flow of the optic disk, visual field defect, peripapillary RNFL, and mGCC was also analyzed.

**Results:**

The mean age of 89 patients with NAION was 56.42 ± 6.81 years (ranging from 39 to 79). The initial RPC whole en face image VD was significantly reduced after acute NAION (≤3 weeks) (F = 45.598, *P* < 0.001) and stabilized from the eighth week onward. Over the course of NAION, the superonasal RPC, superior mGCC, and superotemporal RNFL decreased mostly with time (F = 95.658, 109.787, 263.872, respectively; *P* < 0.001). Maximal correlations were found between superior mGCC and temporosuperior RPC in the NAION phase (*R* = 0.683, *P* < 0.01) and between superonasal RPC and superonasal RNFL (*R* = 0.740, *P* < 0.01). The mean defect was correlated with temporosuperior RPC (*R* = −0.281, *P* < 0.01) and superior mGCC (*R* = −0.160, *P* = 0.012).

**Conclusion:**

Over the course of NAION, OCTA shows a tendency toward change in the retinal capillary plexus of the optic disk. OCTA is proved to be a practical and useful tool for observing papillary perfusion in NAION.

## 1. Introduction

Nonarteritic anterior ischemic optic neuropathy (NAION) is the most common acute optic neuropathy seen in people aged over 50 years. It manifests as a permanent visual field impairment and has unclear pathophysiology (1). It is also the second most common major optic neuropathy after glaucoma, resulting from acute ischemia in the optic nerve (2). Optical coherence tomography–angiography (OCTA) is a new and non-invasive method of vascular mapping that can provide detailed information about the retinal and choroidal vasculature (3, 4).

Recent studies have utilized OCTA in NAION to explore microvascular changes in the optic nerve and macula (5, 6). These studies have shown a decrease in peripapillary capillary density in acute NAION eyes with active disk edema and in chronic NAION eyes as well as an association of the superior temporal region with NAION. The temporal macular vessel density (VD) measured during the acute stage is a significant predictor of final visual outcomes (5, 6). Despite these observations, further studies are warranted to elucidate detailed changes in the blood flow in the optic nerve and their correlation with the visual field in different stages of the disease. Therefore, this study sought to serve as an introduction to the change curve in peripapillary microvasculature and to reveal the relationship between visual function and retinal blood flow and the structure in the corresponding area. To the best of our knowledge, this is the first study to analyze the peripapillary VD by dividing the disease course into five stages and using a relatively large number of patients with NAION.

## 2. Materials and methods

### 2.1. Inclusion and exclusion criteria

This was a prospective observational study. From January 2020 to December 2021, 89 eyes of 89 patients diagnosed with NAION at the Eye Center of the Second Affiliated Hospital of Zhejiang University School of Medicine were included.

The inclusion criteria were as follows: (1) sudden and painless loss of vision, (2) inferior arc-shaped defect of the visual field, (3) diffuse or limited papillary edema and constant linear peripheral hemorrhage, (4) related ocular and systemic risk factors for NAION, (5) a relative afferent papillary defect (+) and/or aberrant visual evoked potential, and (6) the absence of other optic nerve diseases.

The exclusion criteria were as follows: (1) a refractive error >6.0 D of spherical equivalent or 3.0 D of astigmatism;(2) a history of intraocular operation or laser treatment (except for cataract surgery); (3) a history of the retinal, optic, visual pathway, or central nerve systemic disease; (4) the opacity of optic structure leading to inaccurate OCT data (<50) and OCTA data (<7); and (5) poor cooperation with fixation or OCTA examination.

The participants underwent medical optometry, intraocular pressure measurement, slit-lamp microscopic and fundus examinations, fundus photography, visual field test (Octopus 900 24-2), spectral-domain OCT and OCTA, and spectral-domain optical coherence tomography.

Optical coherence tomography–angiography and spectral-domain OCT scans were performed with the AngioVue Imaging System (RTVue XR version 2018.1.0.43; Optovue, Inc, Fremont, CA, USA). Images with a signal strength index <50 in the mGCC map by OCT and <7 in OCTA images were excluded. Using the AngioVue system, 4.5 × 4.5 mm automatic optic disk-centered peripapillary scans were obtained. The whole en face image VD and intra-disk VD were calculated from the 4.5 × 4.5 mm cube scan. We also collected the data of the superficial VD of the radial peripapillary capillaries (RPC) and the retinal nerve fiber layer (RNFL) thickness from the area between the diameter of 2 and 4 mm, which was divided into eight sectors [temporosuperior (TS), superotemporal (ST), superonasal, nasosuperior, nasoinferior (NI), inferonasal (IN), temporoinferior (TI), and inferotemporal (IT)].

Retinal layers were automatically segmented to visualize the superficial vascular plexus in a slab from the internal limiting membrane to 10 μm above the inner plexiform layer (IPL) and the deep vascular plexus from 10 μm above the IPL to 10 μm beneath the outer plexiform layer.

The 6 × 6 mm automatically foveal-centered macular cube was measured to obtain the thickness of the ganglion cell complex (GCC) from the inner limit membrane to the IPL, which included the RNFL, ganglion cell layer, and IPL. The average, superior, and inferior hemispheric GCC thicknesses were recorded for both macular and parafoveal macular areas.

### 2.2. Statistical analysis

The SPSS statistical software version 26 (IBM Corp., Armonk, NY, USA) was used for data analysis. The Shapiro–Wilk test was used to evaluate the data distribution. Normally distributed quantitative data are expressed as mean ± standard deviation and categorical data as percentages (%). Non-normally distributed quantitative data were expressed as median (P25, P75) and compared *via* the Mann–Whitney U-test. The repeated measure analysis of variance for categorical data was used to compare and analyze the differences of each index at different time periods, and the S-N-K method was used to compare every two groups. The Spearman method was used to analyze the correlations of every parameter. A *P*-value of <0.05 was considered to be statistically significant.

### 2.3. Ethical considerations

The research followed the tenets of the Declaration of Helsinki and was approved by the Second Affiliated Hospital of Zhejiang University School of Medicine (approval number: 2020-618). Written consent was obtained from the patients before they participated in this study.

## 3. Results

### 3.1. Patients

A total of 89 patients with NAION (89 affected eyes; average age, 56.42 ± 6.81 years) were included, of whom 52.88% (47) were men and 47.2% (42) were women. Among the affected eyes, 53.9% (48) were right eyes and 46.1% (41) were left eyes. In this group, 23.6% (21) had a self-reported history of hypertension, and 7.9% (7), 5.6% (5), and 4.5% (4) had a history of diabetes mellitus, hematological diseases, and hyperlipidemia, respectively. The best corrected visual acuity quartile at the last follow-up visit was 0.208 (0, 0.478); this was a significant improvement compared with the initial best corrected visual acuity quartile of 0.305 (0.095, 0.681; *P* < 0.001).

### 3.2. Changes in capillary density in the optic nerve

The differences in mean defect (MD), the whole en face image VD, average RPC, and supro-inferior, infero-superior, TS, ST, TI, IT, and NI RPC at each of the five stages were statistically significant (*P* < 0.05 for all; Table 1), whereas differences in IT-RPC were relatively small (*p* = 0.016; *P* < 0.05; Table 1). The RPC reduction decreased from the superior half area to the NI and TI of RPC, and all tended to be stable from the eighth week, except for the IT RPC. The IT-RPC decreased more slightly than the other region throughout the disease course. The IN RPC decreased from the third week (48.91 ± 3.92) to the eighth week (45.76 ± 7.65) and tended to be stable since the eighth week. In our study, the MD had significant differences over time and tended to be stable after the 12th week.

**Table 1:** Longitudinal changes of the visual field and RPC density in patients undergoing serial OCTA at different stages of NAION.

**Index**		**0–3 wk**	**3–8 wk**	**8–12 wk**	**12–24 wk**	**≥24 wk**	***F***	***P***
MD		13.87 ± 4.76^a^	13.32 ± 4.71^a^	12.49 ± 4.34^a^	11.97 ± 3.81^b^	10.68 ± 3.45^b^	7.667	<0.001
RPC density	WI	44.62 ± 2.36^a^	40.71 ± 3.53^b^	38.92 ± 3.87^c^	38.49 ± 4.21^c^	39.42 ± 4.33^c^	45.598	<0.001
	Average	46.63 ± 2.35^a^	41.96 ± 4.22^b^	39.01 ± 4.60^c^	37.90 ± 4.88^c^	39.05 ± 4.93^c^	65.042	<0.001
	TS	47.98 ± 5.01^a^	42.70 ± 7.12^b^	40.16 ± 7.55^c^	39.91 ± 7.99^c^	40.80 ± 6.52^c^	24.798	<0.001
	ST	41.17 ± 4.71^a^	33.35 ± 6.39^b^	29.61 ± 6.91^c^	28.75 ± 6.88^c^	31.23 ± 6.6^c^	59.383	<0.001
	SN	39.42 ± 4.42^a^	31.18 ± 5.04^b^	27.37 ± 5.28^c^	26.95 ± 5.27^c^	26.45 ± 4.95^c^	95.658	<0.001
	NS	43.12 ± 4.26^a^	37.16 ± 7.22^b^	33.57 ± 7.05^c^	32.53 ± 7.31^c^	32.45 ± 7.32^c^	47.699	<0.001
	NI	45.78 ± 4.18^a^	41.67 ± 6.87^b^	36.95 ± 6.99^c^	36.47 ± 7.33^c^	37.52 ± 7.00^c^	31.692	<0.001
	IN	48.91 ± 3.92^a^	47.79 ± 6.46^a^	45.76 ± 7.65^b^	45.12 ± 8.72^b^	44.20 ± 7.97^b^	6.152	<0.001
	IT	55.71 ± 4.99^a^	53.42 ± 6.25^b^	53.00 ± 6.87^b^	52.28 ± 7.99^b^	53.15 ± 7.99^b^	3.126	0.016
	TI	51.79 ± 5.70^a^	48.08 ± 7.25^b^	46.27 ± 8.03^c^	45.54 ± 7.78^c^	49.09 ± 6.94^b^	12.829	<0.001

RPC, radial papillary capillaries; OCTA, optical coherence tomography angiography; NAION, nonarteritic anterior ischemic optic neuropathy; MD, mean defect; wiVD, whole en face image vessel density; idVD, intra-disk image vessel density; TS, temporosuperior; ST, superotemporal; NS, nasosuperior; SN, superonasal; NI, nasoinferior; IN, inferonasal; IT, inferotemporal; TI, temporoinferior.

The super script letters “a, b, and c” indicates that there exists statistically significant difference between the means in the same line if the super script letter is not same, and that there is no statistically significant difference between the means in the same line if the super script letter is same.

**Figure 1:**
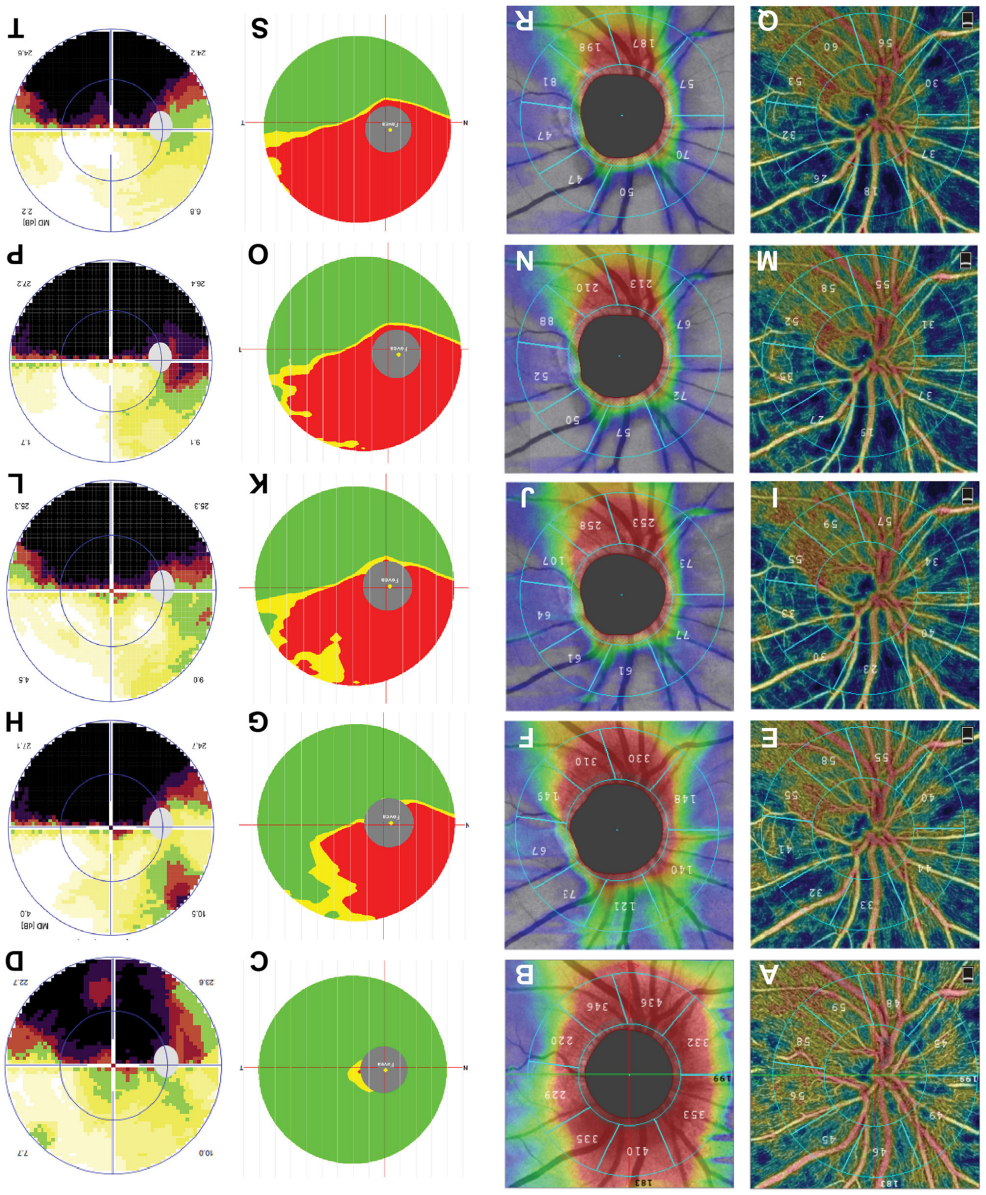
Time course of structural OCT and OCTA findings in a 52-year-old female patient. (a) ≤3 week: **(A–D)**; (b) 3–8 weeks: **(E–H)**; (c) 9–12 weeks: **(I–L)**; (d) 12–24 weeks: **(M–P)**; (e) >24 weeks: **(Q–T)**. The RPC map shows sector atrophy from the acute stage to the chronic stage **(A, E, I, M, Q)**, the pRNFL map shows decreased thickness over time **(B, F, J, N, R)**, and the mGCC map shows the reduction of superior-hemi mGCC over time **(C, G, K, O, S)**. The visual field map shows inferior arc-shaped defects that did not change significantly for better or worse **(D, H, L, P, T)**. OCTA, optical coherence tomography–angiography; RPC; pRNFL, peripapillary retinal nerve fiber layer; mGCC, macular ganglion cell complex.

We also found the typical characteristics in a 52-year-old female patient (Figure 1). She presented with an inferior defect of the visual field (Figures 1D, H, L, P, T) and progressive decrease in the superior RPC (Figures 1A, E, I, M, Q), whole pRNFL (Figures 1B, F, J, N, R), and superior macular GCC (S mGCC) (Figures 1C, G, K, O, S) during the five stages.

### 3.3. Changing tendency of peripapillary RNFL thickness

There were significant differences in eight areas of the peripapillary RNFL (pRNFL) thickness at each of the five stages (*P* ≤0.001 for all; Table 2). The pRNFL thickness tended to decrease in all of the quadrants over time, and a persistent decrease in thickness continued during the observation period.

**Table 2:** Changes of pRNFL in patients with NAION at different stages.

**Index**	**0–3 wk**	**3–8 wk**	**8–12 wk**	**12–24 wk**	**≥24 wk**	***F***	***P***
Average	245.03 ± 51.62^a^	148.59 ± 35.23^b^	98.02 ± 17.20^c^	87.00 ± 15.52^d^	80.20 ± 9.61^e^	326.43	<0.001
TS	192.11 ± 87.67^a^	76.02 ± 19.88^b^	60.15 ± 14.52^c^	59.10 ± 19.80^c^	56.59 ± 10.28^c^	104.57	<0.001
ST	277.46 ± 86.61^a^	123.60 ± 36.73^b^	70.94 ± 16.57^c^	62.38 ± 17.43^d^	60.27 ± 11.98^d^	263.872	<0.001
SN	289.40 ± 88.58^a^	146.06 ± 48.07^b^	80.47 ± 21.90^c^	70.50 ± 18.93^d^	61.68 ± 11.79^e^	252.627	<0.001
NS	254.57 ± 75.44^a^	142.25 ± 45.85^b^	83.64 ± 18.43^c^	74.22 ± 19.50^d^	64.54 ± 12.24^e^	211.287	<0.001
NI	247.31 ± 90.34^a^	153.28 ± 50.54^b^	92.05 ± 22.11^c^	82.61 ± 20.20^d^	72.43 ± 11.82^e^	130.248	<0.001
IN	307.69 ± 87.63^a^	226.38 ± 77.32^b^	149.50 ± 38.22^c^	132.59 ± 32.89^d^	116.55 ± 21.23^e^	119.972	<0.001
IT	263.58 ± 71.05^a^	215.61 ± 72.45^b^	168.88 ± 56.89^c^	144.01 ± 37.89^d^	141.58 ± 27.43^d^	57.698	<0.001
TI	154.10 ± 56.81^a^	99.36 ± 35.37^b^	84.38 ± 35.32^c^	73.76 ± 17.19^d^	75.5 ± 13.09^d^	51.707	<0.001

pRNFL, peripapillary retinal nerve fiber layer; NAION, nonarteritic anterior ischemic optic neuropathy; TS, temporosuperior; ST, superotemporal; NS, nasosuperior; SN, superonasal; NI, nasoinferior; IN, inferonasal; IT, inferotemporal; TI, temporoinferior.

The super script letters “a, b, c, d, and e” indicates that there exists statistically significant difference between the means in the same line if the super script letter is not same, and that there is no statistically significant difference between the means in the same line if the super script letter is same.

### 3.4. Changing tendency of mGCC thickness

There were significant differences in the average mGCC, SmGCC, and inferior macular GCC (I mGCC), and differences were greater in S mGCC than in I mGCC (Table 3). All of them decreased significantly throughout the course of the disease and tended to be stable from the eighth week.

**Table 3:** Changes of mGCC in patients with NAION at different stages.

**Index**	**0–3 wk**	**3–8 wk**	**8–12 wk**	**12–24 wk**	**≥24 wk**	***F***	***P***
Average	97.74 ± 7.11^a^	88.15 ± 8.00^b^	83.04 ± 8.52^c^	83.33 ± 10.07^c^	82.51 ± 6.88^c^	75.925	<0.001
SmGCC	93.08 ± 9.36^a^	77.71 ± 8.78^b^	72.49 ± 9.26^c^	72.57 ± 9.98^c^	72.23 ± 8.03^c^	109.787	<0.001
ImGCC	99.16 ± 15.99^a^	98.48 ± 9.64^a^	93.64 ± 10.71^b^	94.63 ± 12.85^b^	93.25 ± 10.11^b^	3.962	0.004

mGCC, macular ganglion cells; NAION, nonarteritic anterior ischemic optic neuropathy; S mGCC, superior mGCC; ImGCC, inferior mGCC.

The super script letters “a, b, and c” indicates that there exists statistically significant difference between the means in the same line if the super script letter is not same, and that there is no statistically significant difference between the means in the same line if the super script letter is same.

### 3.5. Spearman correlation between time and optic nerve structures

Results showed a negative correlation between time and superior-hemi RPC, pRNFL, and SmGCC. There was no correlation between time and inferior mGCC (Table 4). All of the optic nerve structures decreased in size or became thinner over time.

**Table 4:** Spearman correlation of time and OCTA-recorded retinal structure.

**Index**		***r***	***P***
RPC vessel density	TS	−0.348	<0.01
	ST	−0.479	<0.01
	SN	−0.604	<0.01
	NS	−0.502	<0.01
pRNFL thickness	TS	−0.669	<0.01
	ST	−0.819	<0.01
	SN	−0.835	<0.01
	NS	−0.854	<0.01
mGCC	SmGCC	−0.529	<0.01

OCTA, optical coherence tomography angiography; RPC, radial papillary capillaries; pRNFL, peripapillary retinal nerve fiber layer; TS, temporosuperior; ST, superotemporal; NS, nasosuperior; SN, superonasal; mGCC, macular ganglion cells; S mGCC, superior mGCC.

### 3.6. Spearman correlation between MD with RPC densities and thicknesses of the pRNFL and MGCC

The results of the Spearman correlation between MD and the retinal structures are shown in Table 5. The super-hemi area of RPC had a positive correlation with the corresponding area of pRNFL. MD had a negative correlation with S-mGCC TS RPC, and ST RPC, while MD had a positive correlation with ST, SN, and NS pRNFL.

**Table 5:** Spearman correlation of MD and retinal structure.

**Index**		***r*/*P***	**MD**	**RPC vessel density**				**RNFL thickness**				**S mGCC**
				**TS**	**ST**	**SN**	**NS**	**TS**	**ST**	**SN**	**NS**	
	MD	*r*	1	−0.281^**^	−0.190^**^	0.033	−0.099	0.011	0.134^*^	0.254^**^	0.209^**^	−0.160^*^
		*P*	–	<0.01	0.003	0.603	0.122	0.861	0.036	<0.01	<0.01	0.012
RPC vessel density	TS	*r*		1	0.513^**^	0.301^**^	0.304^**^	0.472^**^	0.438^**^	0.397^**^	0.341^**^	0.683^**^
		*P*		-	<0.01	<0.01	<0.01	<0.01	<0.01	<0.01	<0.01	<0.01
	ST	*r*			1	0.591^**^	0.578^**^	0.498^**^	0.561^**^	0.555^**^	0.514^**^	0.633^**^
		*P*			-	<0.01	<0.01	<0.01	<0.01	<0.01	<0.01	<0.01
	SN	*r*				1	0.676^**^	0.571^**^	0.692^**^	0.740^**^	0.717^**^	0.493^**^
		*P*				-	<0.01	<0.01	<0.01	<0.01	<0.01	<0.01
	NS	*r*					1	0.401^**^	0.510^**^	0.536^**^	0.566^**^	0.434^**^
		*P*					-	<0.01	<0.01	<0.01	<0.01	<0.01
RNFL thickness	TS	*r*						1	0.859^**^	0.686^**^	0.707^**^	0.680^**^
		*P*						-	<0.01	<0.01	<0.01	<0.01
	ST	*r*							1	0.914^**^	0.862^**^	0.709^**^
		*P*							-	<0.01	<0.01	<0.01
	SN	*r*								1	0.892^**^	0.639^**^
		*P*								-	<0.01	<0.01
	NS	*r*									1	0.557^**^
		*P*									-	<0.01
S-mGCC		r										
		P										-

MD, mean defect; RPC, radial papillary capillaries; pRNFL, peripapillary retinal nerve fiber layer; TS, temporosuperior; ST, superotemporal; NS, nasosuperior; SN, superonasal; mGCC, macular ganglion cells; SmGCC, superior mGCC. ^*^means *p* < 0.05; ^**^means *p* < 0.001.

## 4. Discussion

Recent studies have consistently reported a significant reduction of RPCs by OCTA (6, 7). Liu et al. (8) revealed that the superior RPC was lower than the inferior RPC at 1–2/3–6 m in 21 affected NAION eyes. Sönmez et al. (9) showed that RPC was decreased in all regions except the IN region in patients with acute NAION. Moon et al. (6) found that acute-stage temporal peripapillary VD was a significant predictor of final visual outcomes. We divided the disease course into five stages because RPC had the greatest change during the initial 3 weeks and mild change during 4–8 weeks, which tended to stabilize in the eighth week according to our preliminary study.

In our study, we found that all the regions of RPC significantly decreased from the acute to atrophy stage. These reductions tended to slow down from the eighth week, along with a drastic reduction of the ST and superonasal regions, followed by the TS, nasosuperior, TI, and NI regions. The IT RPC had the least change and tended to be stable after the acute stage. These results provide a more detailed overview of regional reduction in patients with NAION from the acute to the chronic stage. A reduction in RPC in the acute stage was caused by the mechanical compression of the edematous optic nerve, coupled with signal attenuation due to tissue edema (10). Kaya (11) and Zhu et al. (12) also found that carotid stenosis with increased carotid wall thickness and decreased blood velocities in the central retinal artery may decrease the blood flow in RPC in patients with acute NAION on color Doppler imaging (CDI). With the resolution of edema and the loss of RNFL after 3 weeks, the decreased metabolic demand after autoregulation also reduced the blood flow in RPC, particularly after 8 weeks. Rougier et al. (13) found that the mean capillary perfusion density and the capillary flux index in the inferior quadrant were lower in the acute NAION eyes. We found that only the IN and NI RPC transiently increased at the acute stage before decreasing. IN and IT RPC showed relatively smaller changes in the five stages. This provided clear evidence for the existence of an autoregulatory phenomenon to compensate for the ischemic process.

In addition, the loss of RNFL and mGCC reportedly occurs at the acute and resolved stages in NAION (5, 11, 14, 15). We observed that all areas of the RNFL became thinner while all the S mGCC, I mGCC, and average mGCC became thinner with disease progression. MD had a negative correlation with sector RPC and S mGCC, but had no correlation with TS RNFL and had a positive correlation with ST, NS, and SN RNFL, which indirectly proved that visual function impairment had a greater association with mGCC than with pRNFL. Our results showed that TS and ST RPC in NAION eyes suddenly decreased at 1–2 weeks, gradually decreased at 3–4 weeks, and tended to stabilize from the eighth week. The TS pRNFL increased at 1–2 weeks before decreasing suddenly from the third week. This may be due to the regression of optic nerve edema during the acute stage (≤3 weeks). We also found a significant correlation between the reduction of TS, ST RPC, and S mGCC. Without a normal control, we could not determine when mGCC and RNFL became thinner than normal or which degenerated first. However, we are the first to show the longitudinal change map for peripapillary RPC, pRNFL, and mGCC. We speculate that this could be supplementary evidence to support the hypothesis that the thinning of mGCC in the macula is secondary to peripapillary axonal degeneration in NAION (16–18).

Liu et al. (8) found that the early damage to the retinal ganglion cell and axons could be detected within 1 week, peaked at 3–6 months, and lasted for 6–12 months. In our study, we found that the reduction of mGCC stabilized from the eighth week onward. According to our previous study, in 29 cases of NAION (19), the damage peaked in the 12th week. In the current study, we had a larger sample size, a more accurate method of statistical analysis, and a longer follow-up duration.

Aghsaei Fard et al. (14) revealed that the superficial and deep macular VDs did not decrease from the acute to the chronic stage in patients with NAION but that superficial macular VD was less than that in healthy controls. They found that RPC, pRNFL, and mGCC decreased from the acute to the chronic stage in 16 patients with NAION (14). We found a similar downward trend with more stages, subarea of observation, and the number of patients. Therefore, based on the gradual reduction in pRNFL SN, ST RPC, and S mGCC, RPC and S mGCC decreased before the third week, which then stabilized after the eighth week because their changes were indirectly secondary to axonal swelling/ischemia and apoptosis of the axon (18). With the loss of axons and thinning of mGCC, metabolic requirements of the optic nerve also decreased. Therefore, the reduction in RPC tended to slow down after the eighth week.

Our study had some limitations. First, although we had a larger number of patients than that in our previous study, our pilot study still only had a small sample size, which may limit the statistical strength of the analysis. Second, although high-quality images were included, the possible influence of optic nerve head swelling at the acute stage of blood flow signal detection cannot be excluded (20). Third, to assess serial changes in the retinal VDs in patients with NAION, longitudinal data collected over time in the same patients with a larger number would be highly desirable. However, we used continuous data from acute to chronic stages, which had been assessed from the NAION onset to the chronic atrophic stage. Finally, we did not have normal and contralateral eyes as a control group, and we did not observe the superficial and deep macrovascular change in the macula and the choroid (21). Therefore, further studies are necessary with a larger number of patients.

## 5. Conclusion

This study was designed to observe the sequence of changes in the retinal structure (RPC, pRNFL, and mGCC) over time in a cohort of patients with NAION. We have provided detailed findings to demonstrate an apparent loss in sector RPC, the whole pRNFL, and SmGCC with the progression of NAION, which is a natural change of the ischemic disease.

## Data availability statement

The original contributions presented in the study are included in the article/supplementary material, further inquiries can be directed to the corresponding author.

## Ethics statement

The studies involving human participants were reviewed and approved by Second Affiliated Hospital of Zhejiang University of Medicine, Clinical Trials Ethics Committee. The patients/participants provided their written informed consent to participate in this study. Written informed consent was obtained from the individual(s) for the publication of any potentially identifiable images or data included in this article.

## Author contributions

All authors listed have made a substantial, direct, and intellectual contribution to the work and approved it for publication.
